# Genetic determinants of obesity: mechanisms, clinical implications, and targeted therapies

**DOI:** 10.1007/s12020-026-04696-3

**Published:** 2026-07-13

**Authors:** Anastasios Serbis, Evanthia Kantza, Michaela Garoufou, Maria Christou, Ekaterini Siomou, Stelios Tigas

**Affiliations:** 1 Department of Pediatrics, School of Medicine, University of Ioannina, Ioannina, 45110 Greece; 2 Department of Endocrinology & Diabetes Center, University of Ioannina, Ioannina, 45110 Greece

**Keywords:** Obesity, Genetics, Polygenic obesity, Syndromic obesity, Monogenic obesity

## Abstract

**Purpose:**

Obesity is a major global health crisis with rising prevalence in both pediatric and adult populations, leading to an increased risk of cardiovascular, metabolic, and other chronic complications affecting all organ systems. A clear understanding of the genetic contributors to polygenic, syndromic, and monogenic obesity is essential for early diagnosis and targeted management.

**Methods:**

Advances in genome-wide association studies (GWAS) and sequencing technologies have greatly expanded our understanding of the genetic alterations underlying this multifaceted disease and have helped in delivering personalized treatment.

**Results:**

The pathogenesis of common, polygenic obesity is related to a complex interplay between genetic susceptibility and environmental factors. Syndromic obesity, a less common form, is characterized by early-onset accompanied by additional features such as developmental delay, dysmorphic traits, and various organ system involvement. The rarest form, monogenic obesity, is characterized by severe early-onset non-syndromic obesity caused by mutations in single genes regulating appetite within the hypothalamus. These monogenic obesity cases, though infrequent, have been instrumental in elucidating key pathways involved in hunger and satiety.

**Conclusion:**

This review provides a comprehensive summary of the most recent findings on the genetic basis of obesity across all age groups, highlighting clinical implications and emerging therapeutic opportunities.

## Introduction

Obesity is a complex, multifactorial disease that has reached epidemic proportions globally. Recent estimates indicate that approximately one in eight people worldwide −nearly one billion adults and 160 million children and adolescents− are living with obesity [1]. Defined by a body mass index (BMI) of ≥ 30 kg/m² in adults or a corresponding BMI for age and sex in children, obesity significantly increases the long-term risk for numerous chronic conditions, including type 2 diabetes, cardiovascular disease, and certain cancers. Although environmental influences such as high-calorie diet and reduced physical activity play a major role, significant evidence points to a substantial genetic component in susceptibility to obesity. Indeed, heritability estimates from twin, family, and adoption studies suggest that 40–70% of the variation in BMI can be attributed to genetic factors [2, 3]. Furthermore, the genetic contribution to BMI appears to vary with age and may have a greater influence during childhood than adult life [4].

During the past two decades remarkable progress has been achieved in uncovering the genetic underpinnings of obesity, driven by advances in genome-wide association studies (GWAS) and next-generation sequencing (NGS). These approaches have identified hundreds of loci, and more than 500 genes potentially involved in energy balance, adiposity, and appetite regulation [5, 6]. Obesity can now be broadly classified into polygenic, syndromic, and monogenic forms, reflecting the spectrum of genetic involvement. Polygenic obesity is by far the most common, resulting from the additive effects of numerous common variants of small effect, modulated by environmental and behavioral influences [7]. Syndromic obesity, though much rarer, is characterized by early-onset accompanied by neurodevelopmental anomalies, dysmorphic features, or multisystem involvement, as seen in conditions such as Prader–Willi, Bardet–Biedl, and Alström syndromes [8]. Monogenic obesity, the rarest form, arises from single-gene mutations, often with high penetrance, typically manifesting as severe early-onset hyperphagia and resultant obesity before the age of five, and even the age of two [6, 9].

Although rare, cases of monogenic obesity have been instrumental in elucidating the key hypothalamic signaling pathways that govern appetite and energy homeostasis, and most notably the leptin–melanocortin axis. Disruptions in this pathway, caused by mutations in genes such as *LEP*, *LEPR*, *POMC*, *MC4R*, and *PCSK1*, result in profound imbalance in hunger and satiety regulation, leading to severe, early-onset obesity [10]. These insights have not only advanced our understanding of the neuroendocrine regulation of body weight but have also opened new avenues for targeted hormonal and receptor-based treatments designed to restore physiological appetite regulation in affected individuals [11].

As the field of obesity genetics continues to evolve, it is increasingly evident that molecular diagnosis plays a pivotal role in personalized care. Genetic testing can facilitate genetic counseling, guide tailored clinical management and help identify individuals who may benefit from emerging targeted therapies, such as recombinant human leptin analogues or melanocortin-4 receptor (MC4R) agonists [12]. In this review, we provide a comprehensive overview of the genetic architecture of obesity, including polygenic, syndromic, and monogenic forms, and discuss the clinical relevance and therapeutic implications of recent genetic discoveries.

## Materials and methods

A comprehensive literature search of the PubMed and Scopus databases for English-language publications, without time restrictions was performed. The search, conducted between December 2024 and March 2025, employed combinations of the terms “obesity,” “genetics,” “polygenic,” “monogenic,” “syndromic,” “children,” “leptin-melanocortin pathway,” and “therapy.” Eligible sources included original research articles, review papers, and meta-analyses. To ensure a thorough overview, relevant case reports—particularly those describing treatment outcomes in rare monogenic obesity—were also included. Additional references were identified through manual screening of bibliographies. The aim was to synthesize the most up-to-date evidence on the genetic underpinnings of obesity in humans and their clinical implications across the lifespan.

## Polygenic obesity

Over the past two decades, GWAS have dramatically advanced our understanding of the genetic basis of polygenic obesity, the most common form of obesity encountered in the general population. In contrast to monogenic obesity, which arises from rare, high-penetrance mutations in single genes, polygenic obesity results from the cumulative effect of hundreds or thousands of common variants, each contributing modestly to variation in the BMI and fat distribution. These common variants, typically single nucleotide polymorphisms (SNPs), influence complex traits by altering gene expression, protein function, or regulatory networks involved in energy balance, appetite regulation, and adipocyte biology. The increased statistical power provided by data analysis from large international consortia −such as GIANT (Genetic Investigation of ANthropometric Traits) and UK Biobank− has enabled the identification of more than 900 SNPs associated with obesity-related traits across diverse populations [13–15].

A landmark discovery in the field was the identification of variants in the *FTO* (Fat Mass and Obesity-associated) gene in 2007 as the first common SNPs robustly associated with BMI [16]. Located in a non-coding region of chromosome 16, *FTO* SNPs are now known to influence energy intake by modulating hypothalamic circuits involved in hunger and satiety. Subsequent studies have revealed that *FTO* risk alleles are associated with increased food intake, preference for energy-dense foods, and diminished satiety responses. For example, the rs9939609 SNP has been consistently identified as a strong genetic predictor of increased obesity risk, while rs1421085 has been associated with greater preference for energy-dense foods, including elevated intake of sugar and fat [17, 18].

Another gene with well-characterized polymorphisms influencing BMI and appetite is *MC4R*. Common SNPs located near or within the *MC4R* gene, such as rs17782313, have been consistently associated with modest increases in BMI and appetite, likely through regulatory effects on gene expression [19]. As with other variants implicated in polygenic obesity, these SNPs are common in the general population and contribute incrementally to the overall obesity risk when combined with variations in other loci. In contrast, rare, loss-of-function mutations in *MC4R* are a well-established cause of monogenic obesity, discussed in detail in the Monogenic Obesity section of this article.

Beyond *FTO* and *MC4R*, several other genes have shown strong and reproducible associations with obesity risk. For example, *BDNF* [20], a gene playing a key role in neuronal plasticity and energy homeostasis, and *SH2B1* [21], a critical mediator of leptin and insulin signaling, are among the loci with robust links to BMI and appetite regulation (Table 1). Notably, many of these genes are expressed in the hypothalamus, underscoring the central role of the central nervous system in regulating energy balance and feeding behavior [5]. However, despite the discovery of hundreds of associated loci, the proportion of BMI variance explained by common variants remains modest, estimated at approximately 5–10%. This phenomenon, often referred to as the “missing heritability,” suggests that the lower proportion of BMI variance explained by genome-wide significant common variants compared with estimates from family-based studies reflects additional genetic and biological complexity, including the contribution of rare variants, epigenetic mechanisms, and gene–gene or gene–environment interactions to individual obesity susceptibility [22, 23].

To quantify the cumulative impact of common variants, researchers have developed polygenic risk scores (PRS), which aggregate risk alleles weighted by their effect sizes [24]. These scores allow for stratification of individuals based on their genetic predisposition to obesity, and high PRS values have been associated with increased risk of severe obesity, insulin resistance, and cardiovascular disease, even from early life. Although not yet integrated into routine clinical practice, PRS holds promise as a tool for personalized prevention, risk prediction, and early intervention in high-risk individuals [25]. Despite this potential, the translation of polygenic risk scores into clinical practice remains limited. Their predictive value is modest when considered alongside established clinical risk factors, and their performance varies significantly across different populations, limiting generalizability. Furthermore, there are currently no standardized thresholds to guide clinical decision-making, and no validated intervention strategies specifically tailored to PRS-defined risk categories. As a result, PRS are not yet incorporated into routine clinical care.

In addition to total adiposity, GWAS have identified distinct genetic determinants of body fat distribution, particularly waist-to-hip ratio (WHR) adjusted for BMI [26]. Notable examples include *LYPLAL1*,* RSPO3*,* VEGFA*,* and HOXC13* [15] (Table 1). These SNPs often show sex-specific effects, with stronger associations observed in women [27, 28]. Importantly, fat distribution is more strongly associated with cardiometabolic complications than BMI alone, underscoring the clinical importance of such genetic traits. Functional follow-up of these loci has highlighted the role of adipocyte subtype differentiation, regional fat depots, and their endocrine functions in metabolic disease risk [29, 30].

Genetic studies have also extended to childhood obesity, revealing substantial overlap with adult obesity loci, but also identifying age-specific effects. For instance, *FTO*, *MC4R*, and *LEPR* variants are associated with life-long adiposity, but certain SNPs may exert stronger effects in childhood than adulthood, or vice versa [31]. A large GWAS meta-analysis by the Early Growth Genetics (EGG) Consortium identified novel loci related to birth weight, early growth trajectories, and childhood BMI [32]. These findings support the concept that genetic influences on obesity begin early in life and may interact with developmental programming, feeding behavior, and environmental exposures to shape long-term risk [33].

Overall, advances in the understanding of the polygenic architecture of obesity have provided important insights into the biological regulation of adiposity and its variability across the lifespan. While current clinical applicability remains limited, genetic data may in the future contribute to improved risk stratification, phenotypic characterization, and understanding of interindividual variability in metabolic complications of obesity and treatment response.

**Table 1:** Key Genes Identified in GWAS to be associated with obesity/BMI, fat distribution, and childhood obesity

Gene	Summary	Category
*FTO *[16]	First common gene robustly associated with BMI, SNP rs9939609	Obesity
*MC4R *[19]	Regulates appetite and energy balance, both monogenic and polygenic effects	Obesity
*NEGR *[107]	Neuronal growth, high brain expression	Obesity
*SH2B1*[21]	Leptin/insulin signaling, deletions linked to syndromic obesity	Obesity
*BDNF *[20]	Affects synaptic plasticity and appetite control	Obesity
*LYPLAL1 *[15]	Associated with waist-hip ratio (WHR), stronger in women	Fat distribution
*RSPO3 *[15]	Regulates adipose tissue development	Fat distribution
*VEGFA* [15]	Involved in angiogenesis, affects fat distribution	Fat distribution
*HOXC13* [15]	Developmental gene, WHR association	Fat distribution
*ADCY3 *[108]	Loss-of-function causes early-onset obesity and increased type 2 diabetes risk in adult life, hypothalamic function	Childhood obesity
*KLF14* [109]	Imprinted gene involved in metabolic regulation and associated with early childhood BMI	Childhood obesity
*OLFM4 *[110]	Associated with early childhood BMI and implicated in gut and immune-related pathways	Childhood obesity

## Syndromic obesity

Syndromic obesity refers to a group of rare, genetically heterogeneous disorders marked by early-onset obesity accompanied by additional abnormalities affecting multiple organ systems. The co-occurrence of severe, treatment-resistant weight gain in early childhood with features such as developmental delay, intellectual disability, or dysmorphic traits should prompt consideration of an underlying genetic syndrome and warrant comprehensive diagnostic evaluation [34]. In a systematic review of genetic syndromes with obesity, Kaur et al. identified 79 distinct syndromes, of which 55 featured obesity as a cardinal characteristic, while the remaining 24 exhibited a higher prevalence of obesity than the general population. Notably, 49 of these syndromes have been mapped to specific chromosomal regions or linked to known causative genes [35]. The introduction of advanced molecular diagnostics, including chromosomal microarray analysis (CMA), targeted gene panels, and whole-exome sequencing (WES), has substantially improved the identification of pathogenic variants in well-established syndromes, such as Prader–Willi (PWS), Bardet–Biedl syndrome (BBS), and Alström syndrome [36].

From a clinical perspective, early recognition of syndromic obesity is essential, as it enables targeted genetic testing, anticipatory screening for multisystem complications (e.g., endocrine, renal, and cardiovascular), and timely initiation of syndrome-specific or pathway-directed therapies within a multidisciplinary care framework [37]. Moreover, it may enable access to emerging targeted therapies, such as MC4R agonists, in selected genetically confirmed cases [38]. In the sections that follow, the three most common forms of syndromic obesity are discussed in greater detail, and Table 2 provides an overview of additional, rarer syndromes associated with obesity.

**Table 2:** Major syndromic causes of pediatric obesity, classified according to estimated prevalence

Syndrome	OMIM	Gene(s) involved	Estimated prevalence	Major clinical features
Prader-Willi syndrome (PWS) [111]	#176270	15q11-q13 deletion/maternal uniparental disomy	1 in 10,000–30,000	Neonatal hypotonia, hyperphagia, severe obesity, hypogonadism, short stature, intellectual disability
Bardet-Biedl syndrome (BBS) [63]	#209900 (genetically heterogeneous, this is the primary phenotype entry)	*BBS1*,* BBS2*,* ARL6 (BBS3)*,* BBS4*,* BBS5*,* MKKS (BBS6)*,* BBS7*,* TTC8 (BBS8)*,* PTHB1 (BBS9)*,* BBS10*,* TRIM32 (BBS11)*,* BBS12*,* MKS1 (BBS13)*,* CEP290 (BBS14)*,* WDPCP (BBS15)*,* SDCCAG8 (BBS16)*,* LZTFL1 (BBS17)*,* BBIP1 (BBS18)*,* IFT27 (BBS19)*,* IFT172 (BBS20)*,* C8orf37 (BBS21)*,* IFT74 (BBS22)*	1 in 100,000–160,000 in general populations (higher in consanguineous populations)	Cone–rod dystrophy, polydactyly, obesity, renal anomalies, cognitive impairment
Alström syndrome [112]	#203800	*ALMS1*	< 1 in 1,000,000	Progressive vision and hearing loss, obesity, insulin resistance, cardiomyopathy
Smith-Magenis syndrome [35]	#182290	17p11.2 deletion (*RAI1*)	1:25,000–50,000	Sleep disturbances, intellectual disability, behavioral problems, obesity
WAGR syndrome [35]	#194072	11p13 deletion (including *WT1* and *PAX6*)	1 in 500,000–1,000,000	Wilms tumor, aniridia, genitourinary anomalies, intellectual disability, obesity
Cohen syndrome [35]	#216550	*VPS13B*	< 1 in 100,000	Obesity, intellectual disability, microcephaly, retinal dystrophy, hypotonia
Simpson-Golabi-Behmel syndrome [35]	#312870 (X-linked form, most common)	*GPC3*	< 1 in 100,000, exact prevalence unknown	Pre- and postnatal overgrowth, obesity, coarse facial features, increased tumor risk
Carpenter syndrome [35]	#201000	*RAB23*	< 1 in 1,000,000	Craniosynostosis, polydactyly, obesity, developmental delay
Albright Hereditary Osteodystrophy [113]	#103580 (: overlaps with pseudohypoparathyroidism spectrum)	*GNAS*	rare; prevalence unclear	Short stature, obesity, round face, subcutaneous calcifications, resistance to PTH ± other hormones

Abbreviations: OMIM, Online Mendelian Inheritance in Man.

### Prader-Willi syndrome

PWS is a complex neurodevelopmental disorder and the most common genetic cause of syndromic obesity. It affects approximately 1 in 10,000 to 29,000 individuals, with equal prevalence across sexes and ethnic backgrounds [39]. Population-based data indicate an estimated survival of approximately 87% to 35 years, with cardiovascular and respiratory diseases representing the leading causes of death [40]. The condition results from the loss of expression of paternally inherited genes on chromosome 15q11.2–q13, most commonly due to a paternal deletion (~ 70%), maternal uniparental disomy (~ 25%), or imprinting defects (~ 5%) [41]. These genetic abnormalities impair hypothalamic development and function, leading to the hallmark features of PWS: hypotonia and poor feeding in early infancy, followed by hyperphagia and progressive obesity, short stature secondary to growth hormone deficiency, hypogonadotropic hypogonadism, developmental delays, and behavioral issues, including temper outbursts and obsessive–compulsive traits [39]. The trajectory of obesity in PWS is unique and follows a distinct, age-related progression in multiple nutritional phases, which reflect changes in feeding behavior, energy balance, and metabolic risk over time [42]. The pathogenesis of obesity in PWS is multifactorial, involving defective satiety signaling, reduced energy expenditure, and neuroendocrine dysregulation. Notably, individuals with PWS exhibit markedly elevated fasting and postprandial plasma ghrelin levels, even before hyperphagia begins, further contributing to the drive to eat and excessive weight gain [43]. In addition to hyperphagia and obesity, PWS is associated with important metabolic complications, including insulin resistance, dyslipidemia, metabolic syndrome, and type 2 diabetes mellitus, particularly in adolescents and adults with severe obesity [44–46].

While there is currently no cure for PWS, multidisciplinary management is essential and includes strict dietary supervision, environmental control of food access, behavioral interventions, and long-term multidisciplinary follow-up [47]. Pharmacological management includes growth hormone therapy, which improves body composition, increases lean mass, and enhances physical function, as well as sex hormone replacement at the appropriate age [48]. In addition, several agents targeting hyperphagia and obesity have been explored, including GLP-1 receptor agonists, diazoxide choline, and setmelanotide [49, 50]. Regarding the potential role of incretin-based therapies, such as exenatide, liraglutide, and more recently semaglutide, data remains limited, largely deriving from case reports or small case series, with inconsistent results [51–54]. Diazoxide choline controlled/extended-release has shown reductions in hyperphagia and favorable effects on body composition and cardiometabolic markers in clinical studies [55–57], and diazoxide choline extended-release tablets were approved by the FDA in 2025 for treatment of hyperphagia in adults and pediatric patients aged ≥ 4 years with PWS [58]. However, glycemic monitoring is important because hyperglycemia has been reported during diazoxide choline treatment [59]. In addition, setmelanotide, a melanocortin-4 receptor agonist, has also been evaluated in clinical trials in PWS, albeit with limited and variable effects on hyperphagia and weight outcomes in this population [60].

### Bardet–Biedl syndrome

BBS is a rare, multisystem autosomal recessive disorder classified among the ciliopathies, a group of conditions caused by defects in the structure or function of cilia [61]. It is clinically characterized by a combination of early-onset obesity, retinal dystrophy, typically presenting as a progressive cone–rod dystrophy, postaxial polydactyly, renal anomalies, hypogonadotropic hypogonadism, and intellectual disability [62]. Although precise mortality estimates are lacking, available evidence suggests that life expectancy in BBS is reduced, primarily due to progressive renal disease and cardiometabolic complications [63]. BBS results from biallelic mutations in any of > 25 identified BBS genes, most of which encode proteins involved in the assembly or function of the BBSome complex critical for primary cilium activity. Such mutations can be missense, nonsense, deletions, insertions or duplications [64]. Primary cilia are essential for several cellular signaling pathways, including those involved in leptin and MC4R signaling in the hypothalamus. Disruption of these pathways contributes to hyperphagia, impaired satiety, and dysregulated energy homeostasis in BBS [65]. Obesity typically begins in early childhood and may progress to severe obesity with insulin resistance or type 2 diabetes during adolescence. Visual deterioration due to rod–cone dystrophy (a retinitis pigmentosa-like degeneration) generally begins in childhood and often leads to blindness by early adulthood. Renal involvement, ranging from structural abnormalities to progressive chronic kidney disease, represents a major cause of morbidity and mortality in affected individuals [64].

There is currently no curative therapy for BBS. Treatment is supportive and multidisciplinary, focusing on nutritional and behavioral interventions, ophthalmologic surveillance, renal function monitoring, and hormonal replacement therapy for hypogonadism. Importantly, setmelanotide has demonstrated efficacy in reducing hunger and promoting weight loss in patients with genetically confirmed BBS by bypassing the defective melanocortin pathway and has been approved by both the Food and Drug Administration (FDA) and European Medicines Agency (EMA) for this indication [66]. In addition, GLP-1 receptor agonists such as semaglutide have recently been explored, suggesting a potential adjunctive therapeutic role, although evidence remains limited to case-based data [67]. Early diagnosis, preferably through molecular genetic testing, is critical to enable anticipatory guidance, targeted management, and access to emerging precision therapies.

### Alström syndrome

Alström syndrome is a rare autosomal recessive disorder and a well-recognized cause of syndromic obesity, associated with reduced life expectancy, often due to progressive multi-organ failure, particularly cardiomyopathy, renal disease, and metabolic complications. It is caused by biallelic mutations in the *ALMS1* gene, located on chromosome 2p13. This gene encodes a protein whose precise function remains incompletely understood but is thought to be involved in ciliary structure and intracellular trafficking processes [68]. Although the clinical phenotype overlaps with other ciliopathies such as BBS, Alström syndrome exhibits several distinctive clinical features [69]. Key manifestations include severe early-onset obesity, insulin resistance progressing to type 2 diabetes, cone–rod retinal dystrophy (leading to progressive visual loss), sensorineural hearing loss, dilated or restrictive cardiomyopathy, hepatic steatosis or fibrosis, pulmonary fibrosis, and progressive renal impairment [70]. In contrast to BBS, polydactyly and intellectual disability are absent or mild, providing important clues for differential diagnosis. Obesity typically begins in infancy and is associated with hyperphagia and features of the metabolic syndrome [71].

There is currently no disease-specific therapy for Alström syndrome. Management is supportive and multidisciplinary, focusing on metabolic control, preservation of organ function, and supportive management of visual and hearing impairment (e.g., visual and auditory aids) [72]. Early dietary intervention and regular metabolic surveillance are essential to reduce obesity-associated morbidity. Although setmelanotide has demonstrated efficacy in Bardet–Biedl syndrome and other genetic obesity disorders involving impaired melanocortin signaling, evidence in Alström syndrome remains limited and inconclusive, with only a small number of Alström patients included in the pivotal BBS/Alström trial [66]. In parallel, real-world data from a large cohort of patients with Alström syndrome indicate that GLP-1 receptor agonists may confer clinically meaningful improvements in weight and glycemic control, supporting their potential off-label use in this monogenic syndromic obesity context [73]. Timely molecular diagnosis is critical for guiding clinical care, facilitating systemic monitoring, enabling genetic counseling, and providing access to emerging precision therapies.

## Monogenic Obesity

**Fig. 1:**
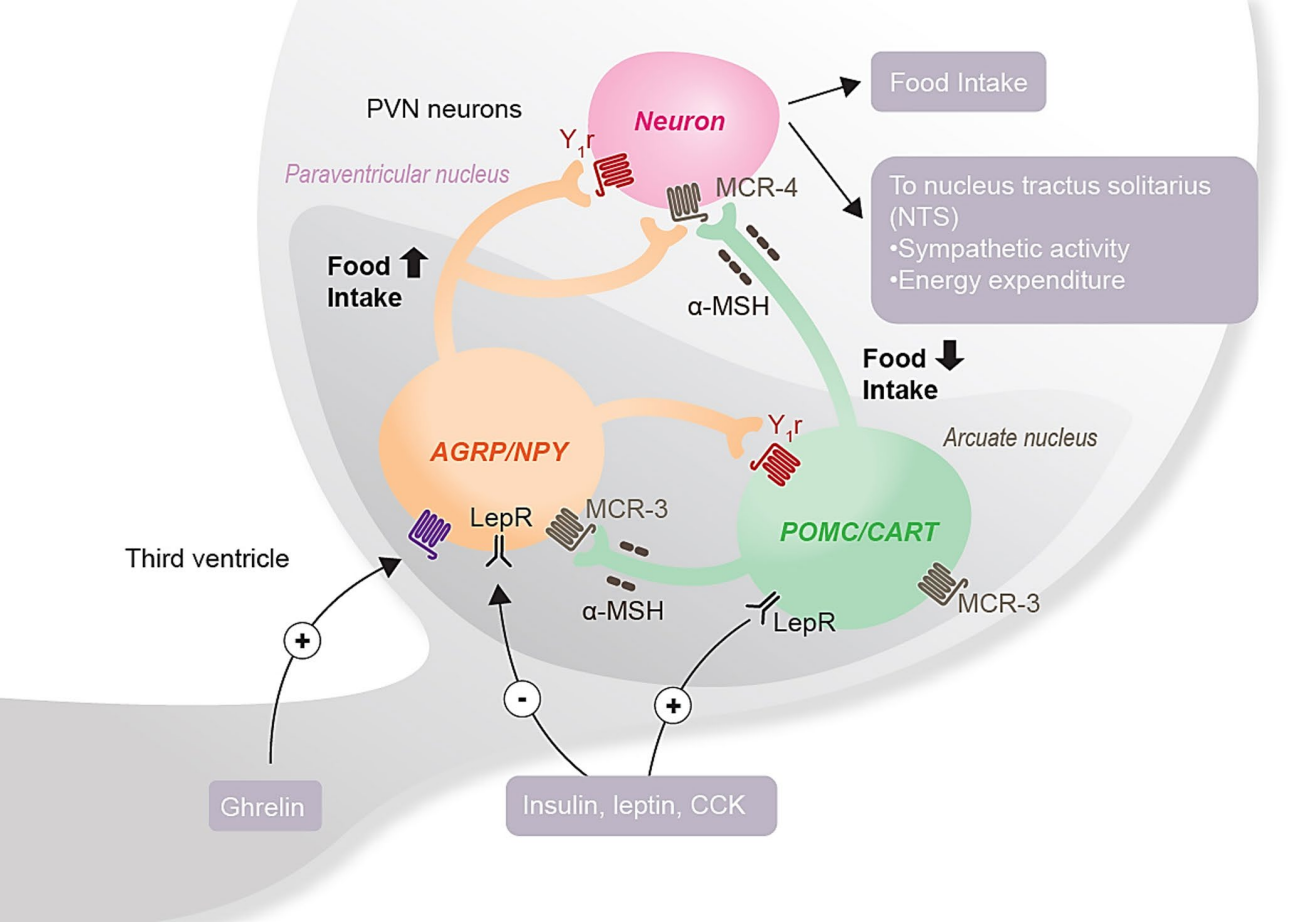
Schematic representation of neuronal populations within the arcuate nucleus and the paraventricular nucleus (PVN) that are mainly responsible for the hypothalamic appetite regulation. Two key populations —anorexigenic POMC/CART neurons and orexigenic AGRP/NPY neurons— respond to peripheral metabolic signals such as leptin, insulin, ghrelin, and cholecystokinin (CCK). Leptin and insulin act via leptin receptors (LepR) to activate POMC/CART neurons, leading to the release of alpha-melanocyte-stimulating hormone (α-MSH), which activates melanocortin receptors (MC3R and MC4R) on PVN satiety neurons. At the same time, they inhibit the hunger pathway of AGRP/NPY neurons, ultimately suppressing food intake and decreasing resting energy expenditure. Conversely, ghrelin stimulates AGRP/NPY neurons which antagonize MC4R signaling, increasing appetite and promoting feeding. Signals from the PVN are transmitted to the nucleus tractus solitarius, where they influence sympathetic tone and energy expenditure. **Abbreviations**: AGRP, agouti-related peptide; α-MSH, alpha-melanocyte-stimulating hormone; CART, cocaine- and amphetamine-regulated transcript; CCK, cholecystokinin; LepR, leptin receptor; MC3R/MC4R, melanocortin 3/4 receptor; NPY, neuropeptide Y; NTS, nucleus tractus solitarius; POMC, pro-opiomelanocortin; PVN, paraventricular nucleus

Over the past two decades, advances in genetic technologies, including candidate gene sequencing, WES, and GWAS. have facilitated the identification of rare, high-penetrance mutations in genes involved in hypothalamic appetite regulation, such as *LEP*, *LEPR*, *POMC*, *PCSK1*, and *MC4R* [74, 75].These discoveries, often made in individuals from consanguineous families presenting with early-onset, severe obesity unresponsive to conventional interventions, have underscored the pivotal role of the leptin-melanocortin signaling pathway in human energy homeostasis (Fig. 1). More recently, additional genes, such as *SIM1*,* have* been implicated in severe obesity, primarily through their effects on hypothalamic development or downstream intracellular signaling mechanisms [34, 74]. Importantly, the identification of monogenic obesity has direct therapeutic implications, as several of these conditions are now amenable to targeted treatments, highlighting the critical role of early genetic diagnosis in guiding personalized management. In the following sections, the more frequently encountered forms of monogenic obesity are discussed in detail, while a broader overview of implicated genes is provided in Table 3.

### *LEP* and *LEPR* genes

Leptin is a hormone primarily secreted by subcutaneous white adipocytes, with lesser contributions from brown adipose tissue, the placenta, stomach, and skeletal muscle. After crossing the blood–brain barrier, leptin binds to its receptor (LEPR) on presynaptic GABAergic neurons in the arcuate nucleus of the hypothalamus, activating proopiomelanocortin (POMC) and cocaine- and amphetamine-regulated transcript (CART) neurons [76]. In parallel, it inhibits the orexigenic pathway mediated by neuropeptide Y (NPY) and agouti-related peptide (AgRP) neurons [77]. In this way, leptin plays a central role in energy homeostasis by promoting satiety, reducing appetite, and increasing energy expenditure. The amount of leptin secreted is directly proportional to the total adipose tissue mass, serving as a peripheral signal to the central nervous system regarding the body’s energy reserves. The leptin gene (*LEP*) is located on chromosome 7q32.1. Mutations in *LEP* are rare and typically inherited in an autosomal recessive manner, most commonly observed in consanguineous families [78, 79]. They include missense, nonsense, frameshift, and splice-site mutations [80]. Affected individuals present clinically with normal birth weight but rapidly gain weight within the first months of life due to severe hyperphagia, leading to marked obesity by early childhood. Additional features include recurrent infections, obesity-related metabolic complications (e.g., hypertension, dyslipidemia, hyperglycemia), and hypogonadotropic hypogonadism [79]. Biochemically, these individuals exhibit undetectable or extremely low serum leptin levels. Subcutaneous administration of recombinant human leptin (metreleptin) has been shown to significantly reduce hyperphagia and body weight, normalize metabolic parameters, and restore function of the hypothalamic–pituitary–gonadal axis [81, 82].

Patients with mutations in the leptin receptor gene (LEPR), located on chromosome 1p31.3, may present with a clinical phenotype similar to congenital leptin deficiency, but with normal or elevated circulating leptin levels. Despite adequate leptin production, these individuals exhibit impaired leptin signaling due to dysfunctional leptin receptors, resulting in early-onset obesity and pronounced hyperphagia. LEPR mutations are inherited in an autosomal recessive manner and include missense, nonsense, frameshift, and splice-site variants, as well as larger deletions or insertions [83, 84]. Unlike congenital leptin deficiency, treatment with recombinant leptin is ineffective. However, setmelanotide, an MC4R agonist, has demonstrated efficacy and safety in patients with LEPR deficiency, bypassing the defective leptin signaling pathway [85].

**Table 3:** Well-established, non-syndromic monogenic causes of obesity in humans

Gene	OMIM	Protein / Pathway	Prevalence; Major clinical features	Therapeutic approach
*LEP *[114]	#164160	Leptin	Extremely rare (< 1:1,000,000); Severe early-onset obesity, hyperphagia, hypogonadism, immune dysfunction	Metreleptin replacement, hormonal replacement
*LEPR *[115]	#601007	Leptin receptor	Extremely rare (< 1:1,000,000); Severe early-onset obesity, hyperphagia, hypogonadism, immune dysfunction	Setmelanotide, hormonal replacement
*POMC *[116]	#609734	Precursor to α-MSH, ACTH	Extremely rare (< 1:1,000,000); Obesity, adrenal insufficiency, red hair, hypopigmentation	Setmelanotide, glucocorticoid replacement
*PCSK1 *[117]	#162150	Prohormone convertase	Extremely rare (< 1:1,000,000); Obesity, hypoglycemia, diarrhea, multiple hormone deficiencies	Setmelanotide, hormone replacement
*MC4R *[118, 119]	#155541	Melanocortin-4 receptor	Most common monogenic obesity (~ 2–5% of severe early-onset obesity); Early-onset obesity, hyperphagia, tall stature	Lifestyle intervention, GLP-1 analogs, setmelanotide in selected cases
*SIM1 *[120]	#603128	Hypothalamic development	Very rare (exact prevalence unknown); Hyperphagia, obesity, mild neurodevelopmental symptoms	Supportive care, behavioral therapy
*SH2B1 *[121]	#608937	Leptin and insulin signaling	Rare (< 1% of severe obesity; often CNVs); Severe obesity, insulin resistance, behavioral problems	Metformin (off-label), setmelanotide (off-label), behavioral interventions
*MRAP2 *[122]	#611488	Modulates MC4R signaling	Very rare (exact prevalence unknown); Severe early-onset obesity, hyperphagia	Supportive; MC4R agonists under investigation
*BDNF* [123]	#113505	Neurotrophin for satiety signaling	Very rare (exact prevalence unknown); Obesity, hyperphagia, cognitive deficits	Supportive, no targeted therapy
*NTRK2* [124]	#600456	BDNF receptor	Extremely rare; Severe obesity, developmental delay, insatiable hunger	Supportive care

Abbreviations: ACTH, adrenocorticotropic hormone; α-MSH, alpha-melanocyte-stimulating hormone; BDNF, brain-derived neurotrophic factor; GLP-1, glucagon-like peptide-1; MC4R, melanocortin 4 receptor; OMIM, Online Mendelian Inheritance in Man.

### *POMC* gene

Further downstream in the satiety signaling pathway, pro-opiomelanocortin (POMC)-producing neurons are located in the arcuate nucleus of the hypothalamus. The *POMC* gene, situated on chromosome 2p23.3, encodes a precursor polypeptide that undergoes post-translational cleavage to produce several biologically active peptides, including adrenocorticotropic hormone (ACTH), α-melanocyte-stimulating hormone (α-MSH), and β-endorphin. These peptides are critical regulators of energy homeostasis, adrenal function, and pigmentation. Biallelic defects in *POMC* include missense, nonsense, and frameshift mutations leading to a rare autosomal recessive disorder characterized by severe early-onset obesity, central adrenal insufficiency, and, in many cases, pale skin and red hair, especially in individuals from populations where such pigmentation is uncommon. These clinical features reflect impaired production of melanocortins (primarily α-MSH) and ACTH [86, 87]. Adrenal insufficiency may present during the neonatal period with hypoglycemia, hypotension, or seizures, necessitating prompt recognition and lifelong glucocorticoid replacement therapy. While delayed or abnormal pubertal development has been reported in some individuals with *POMC* deficiency, central hypogonadism is more consistently associated with *LEPR* or *MC4R* mutations rather than with *POMC* mutations [88]. As POMC neurons lie downstream of leptin receptors, individuals with *POMC* mutations do not respond to recombinant leptin therapy. However, setmelanotide, a selective MC4R agonist, has provided an effective and well-tolerated treatment option for both children (from the age of 2) [89] and adults [85, 90] with genetically confirmed POMC deficiency.

### *PCSK1* gene

Proprotein convertase 1/3 (PC1/3) is an enzyme essential for the post-translational processing of several prohormones into their active forms, including POMC, proinsulin, proglucagon, and prothyrotropin [91]. Homozygous loss-of-function mutations in the relevant *PCSK1* gene, located on chromosome 5q15, lead to a rare form of non-syndromic monogenic obesity. Clinically, affected individuals often present in infancy with severe malabsorptive diarrhea, failure to thrive, and hypoglycemia due to impaired proinsulin processing [92]. As children grow, they develop severe early-onset obesity associated with hyperphagia [93]. Since PC1/3 is involved in the processing of various hormones, additional endocrine abnormalities can gradually appear including central adrenal insufficiency, hypogonadotropic hypogonadism, central hypothyroidism, and growth hormone deficiency [92]. Further, due to extreme obesity, some individuals may develop early-onset type 2 diabetes during adolescence or adulthood. Regarding treatment, both FDA and EMA have approved the use of setmelanotide to reduce hunger and promote weight loss by bypassing the impaired POMC processing [85, 94]. Management also includes appropriate hormone replacement therapy (e.g., hydrocortisone, levothyroxine, sex steroids), nutritional support in infancy, and standard treatment for diabetes and obesity complications. Due to the multisystem nature of the deficiency, care requires a multidisciplinary team including endocrinologists, gastroenterologists, and dietitians [92].

### *MC4R* gene

The MC4R plays a central role in regulating appetite and energy expenditure, functioning as the final effector in the leptin–melanocortin signaling pathway. It is a G protein–coupled receptor expressed primarily in the hypothalamus and is activated by α-MSH, a peptide derived from POMC. The *MC4R* gene, located on chromosome 18q21.32, mediates the anorexigenic effects of melanocortins by promoting satiety and energy expenditure while suppressing food intake. Given its critical role, it is not surprising that loss-of-function mutations in *MC4R* represent the most common monogenic form of obesity, accounting for approximately 2–5% of all severe obesity cases [95, 96]. MC4R mutations are usually inherited in an autosomal dominant pattern, although both heterozygous and homozygous or compound heterozygous variants have been reported [97]. Homozygous individuals generally exhibit a more severe phenotype. Affected individuals typically present with severe hyperphagia and early-onset obesity, often accompanied by accelerated linear growth, hyperinsulinemia, insulin resistance, and increased fat and lean mass. In contrast to POMC or leptin pathway deficiencies, adrenal insufficiency and hypogonadotropic hypogonadism are not characteristic features, as both ACTH production and leptin-dependent GnRH regulation remain intact [98]. Reported mutations include missense, nonsense, and frameshift variants, many of which impair receptor expression, membrane trafficking, or ligand binding [99]. Treatment of MC4R deficiency remains challenging and lifestyle interventions have limited efficacy in these patients. In most cases, the MC4R agonist setmelanotide is ineffective, as it cannot activate a completely nonfunctional receptor. Its utility appears limited to individuals with partial loss-of-function mutations, in whom some receptor activity is preserved. Recently, GLP-1 receptor agonists have been explored as a therapeutic option in this population, with some success. These agents seem to act both on the melanocortin pathway and independently. More specifically, they exert their anorexigenic effects partly through inhibition of the NPY/AgRP hunger pathway, offering a promising adjunct or alternative approach in the management of MC4R-related obesity [100, 101].

### *SIM1* gene

Neurons in the paraventricular nucleus (PVN) of the hypothalamus are critical higher-order regulators of energy balance that integrate signals from peripheral and central pathways, including the melanocortin system, to modulate appetite. Given their central role in satiety signaling, it is not surprising that genetic mutations affecting hypothalamic development can manifest as early-onset obesity. One such example is the *SIM1* (Single-minded 1) gene, which is located on chromosome 6q16.3 and encodes a transcription factor essential for the differentiation and survival of PVN neurons [102]. Haploinsufficiency of SIM1 in humans leads to a rare form of non-syndromic monogenic obesity, characterized by disruption of hypothalamic development and consequent hyperphagia and severe obesity beginning in early childhood [103]. The phenotype often resembles that of MC4R deficiency, but in some cases, it may be more severe. In addition to obesity, some individuals may present with mild neurodevelopmental or behavioral abnormalities, although these are generally less pronounced than those seen in syndromic conditions such as Prader–Willi syndrome [104]. Endocrine abnormalities, including hypogonadotropic hypogonadism and central hypothyroidism, have been observed in a subset of patients, reflecting a possible broader PVN dysfunction [105]. Currently, there is no targeted pharmacotherapy approved specifically for SIM1-related obesity. Since SIM1 functions downstream of leptin and MC4R, MC4R agonists such as setmelanotide are largely ineffective in these patients. As such, treatment is supportive, emphasizing structured dietary interventions, behavioral therapy, and close monitoring for metabolic comorbidities [106]. Ongoing research continues to explore the role of PVN neurons in energy homeostasis and the potential for therapeutic intervention at other nodes within the hypothalamic regulatory network.

## Conclusions

Genetic research has significantly advanced our understanding of obesity, revealing a spectrum from common polygenic forms to rare syndromic and monogenic cases. While polygenic obesity is influenced by the presence of numerous common variants, rare mutations in genes like *LEP*, *LEPR*, *MC4R*, and *POMC* have illuminated key appetite-regulating pathways. Early genetic diagnosis is crucial, particularly in severe or syndromic cases, as it can guide targeted therapies such as setmelanotide or metreleptin. Overall, integrating genetic diagnosis into routine clinical practice has the potential to transform the management of obesity from a generalized approach to a precision medicine model.

## Data Availability

No datasets were generated or analysed during the current study.
